# Software bug report dataset from Eclipse projects

**DOI:** 10.1016/j.dib.2025.112016

**Published:** 2025-09-05

**Authors:** Noelia Lopez-Duran, David Romero-Organvidez, Fermín L. Cruz, David Benavides

**Affiliations:** I3US, Department of Computer Languages and Systems, Universidad de Sevilla, Spain

**Keywords:** Bug report, Bug extraction, Software, Mining software repository, Bug report extraction tool

## Abstract

In recent decades, the analysis of data from software projects — including source control systems, defect tracking systems, and code review repositories — has greatly improved our understanding of software development and its evolution. However, obtaining this information can be time-consuming, and the extracted data is not always well-maintained. This paper introduces an extensive dataset generated from Bugzilla repositories, focusing on key products from the Eclipse bug-tracking system. This dataset addresses the need for up-to-date data in existing repositories, preserving crucial historical information that may be lost due to the transition from Bugzilla to newer bug-tracking systems like Jira or GitHub Issues. Our dataset includes 301,378 bug reports along with all related information, organised into different folders that indicate the project in which the bug was filed. Additionally, we present a custom and lightweight Command Line Interface (CLI) tool designed to efficiently extract detailed information from Bugzilla repositories, automating data collection across various Bugzilla instances. The dataset and tool can be utilized for defect prediction, software maintenance, and evolutionary analysis. To the best of our knowledge, this is the largest, most complete, and up-to-date dataset of Eclipse bug reports available.

Specifications Table

**Table:** 

Subject	Computer Sciences
Specific subject area	Software bug reports from various Eclipse projects
Type of data	Raw Table (.csv format)
Data collection	The dataset was generated using a custom Command Line Interface (CLI) tool specifically designed for extracting comprehensive information from Bugzilla repositories. This tool automates the process of querying Bugzilla's APIs, ensuring a consistent and thorough collection of data across different instances of Bugzilla.
Data source location	The data was collected from *Eclipse’s Bugzilla*, but we also provide data obtained from *Mozilla’s Bugzilla* as it was collected to validate the CLI tool. The described datasets are available at: Eclipse Dataset: https://doi.org/10.5281/zenodo.14229936 Mozilla Dataset: https://doi.org/10.5281/zenodo.14229871
Data accessibility	Repository name: Zenodo Data identification number: https://doi.org/10.5281/zenodo.14229936, https://doi.org/10.5281/zenodo.14229871 Direct URL to data: https://zenodo.org/records/14229937, https://zenodo.org/records/14229872
Related research article	Lopez-Duran, N., Romero-Organvidez, D., Cruz, F. L., & Benavides, D. (2025). Configuration bugs classification using LLMs and encoders ACM Reference Format. *29th ACM International Systems and Software Product Line Conference - Volume A (SPLC-A ’25), September 1–5, 2025, A Coruña, Spain, 1*, 11. https://doi.org/10.1145/3744915.3748477

## 1 Value of the Data

• Eclipse datasets present some challenges such as the timeliness of the data presented. Since many projects have migrated from Bugzilla to other issue-tracking systems, historical data risks being lost. Many existing datasets are outdated [[1], [2], [3], [4], [5]], limiting their usefulness for studies comparing the early stages of a project with more mature stages /projects, studies that need to evaluate bug reports over time, or even studies seeking to analyse long-term trends in software development. This dataset overcomes that limitation by capturing bug reports from the earliest Bugzilla records up to November of 2024, allowing a large historical view.
• Unlike the Bugzilla CSV dump, which only includes a limited set of attributes, and some other published dataset [[1], [2], [3], [4]], this dataset includes the full range of attributes common to every Bugzilla instance. Our dataset is obtained through a systematic methodology using a CLI-based tool, Issuex,^1^ which ensures a complete and up-to-date collection of bug reports. The dataset captures all available attributes, including bug history, resolution changes, comments, and attachments, offering a richer source of information compared to raw extractions. This level of completeness allows researchers to have a wide variety of data available for any given study or to filter the information to work only with the attributes needed for their study.
• This dataset is specifically curated to include the most relevant and either active or inactive Eclipse projects, ensuring that researchers work with data from the most impactful components of the ecosystem. The selection includes major Eclipse products such as Platform, JDT, CDT, PDE, Equinox, BIRT, Mylyn, TPTP, and Papyrus which are crucial to the Eclipse community. By focusing on these key projects, the dataset provides meaningful insights into widely adopted software components, making it more valuable for research in defect prediction, software evolution, and maintenance**.**
• Our dataset provides up-to-date, historical bug reports, allowing researchers to analyse software evolution, defect trends and maintenance practices over time. Its structured format supports seamless integration with data mining and automated analysis tools, facilitating large-scale software quality studies, developer collaboration and problem-solving strategies. With detailed bug histories, resolution changes, comments and attachments, the dataset is ideal for training and benchmarking predictive and troubleshooting models. In addition, its inclusion of multiple Eclipse products allows for comparative analysis, helping researchers identify the best practices and trends in different software development and maintenance projects.
• Beyond its primary use in software engineering research, the dataset’s richness enables broader analyses. Its large temporal coverage, detailed bug information and logs of changes (status update, reassignments, and resolutions) make it possible to study how open-source development communities evolve and interact over time. The inclusion of developer information and comments allows the exploration of potential collaboration patterns or communication dynamics. In addition, with this temporal depth, productivity trends and bug life-cycle analysis can be performed. Analysing temporal factors, we can identify bottlenecks or evaluate the impact of open-source team structures on bug resolution time. This dataset also enables studies on workload distribution, expertise evolution, and sentiment analysis of technical discussions. These insights can enhance bug triaging and task allocation, especially in communities with diverse developer profiles. By understanding how contributions are distributed and how developer knowledge and communication evolve, teams can improve coordination, prioritize issues, and optimize project management.
• Similar datasets have already demonstrated their value in advancing software engineering research. For example, *C. Weiss* et al. [6] used a reduced and limited dataset, similar in structure to ours, to predict bug resolution time, a critical factor in project planning. Likewise, other studies [7,8] have relied on comparable datasets to categorize and prioritize bug reports, an area that remains important in software engineering. With its richer attributes, extensive temporal coverage, and detailed change histories, our dataset provides a stronger foundation for predictive models and more effective automated bug triaging strategies.

## 2 Background

Over the last few decades, the usage of information available about software systems and projects, such as source control systems, defect tracking systems, or even code review repositories, has had a significant impact on the way we understand software development and its evolution [9,10] .This information has allowed the development of bug classification systems, prediction systems for project development, and even automatic code review, thus facilitating project planning and control [6,[11], [12], [13]] However, obtaining this information is often time-consuming, and the extracted data is only sometimes maintained, leading to gaps and inconsistencies that interfere with the research and development efforts [1,14,15].

Although there are existing datasets for some Eclipse projects, there is a notable lack of attribute richness and up-to-date data. The available datasets [[1], [2], [3], [4], [5]] often fall short in either data completeness or timeliness compared to Bugzilla. This highlights the urgent need for a dataset like ours that provides thorough and current information.

## 3 Data Description

The dataset provides a structured collection of bug reports from The Eclipse Foundation extracted from its bug tracking system, Bugzilla. It consists of bug reports from the earliest Bugzilla records up to November of 2024, with a total of **301,378 bug reports** originating from various Eclipse projects. The data is systematically organized into folders and subfolders, ensuring easy navigation and retrieval.

**Fig. 1:**
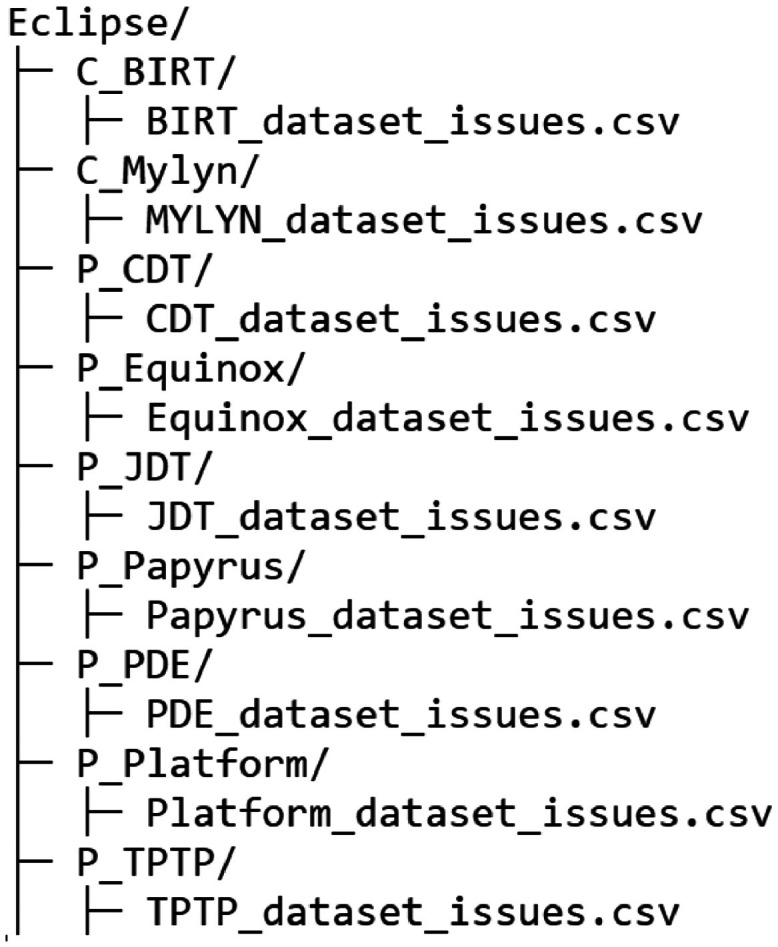
Dataset structure.

**Fig. 2:**
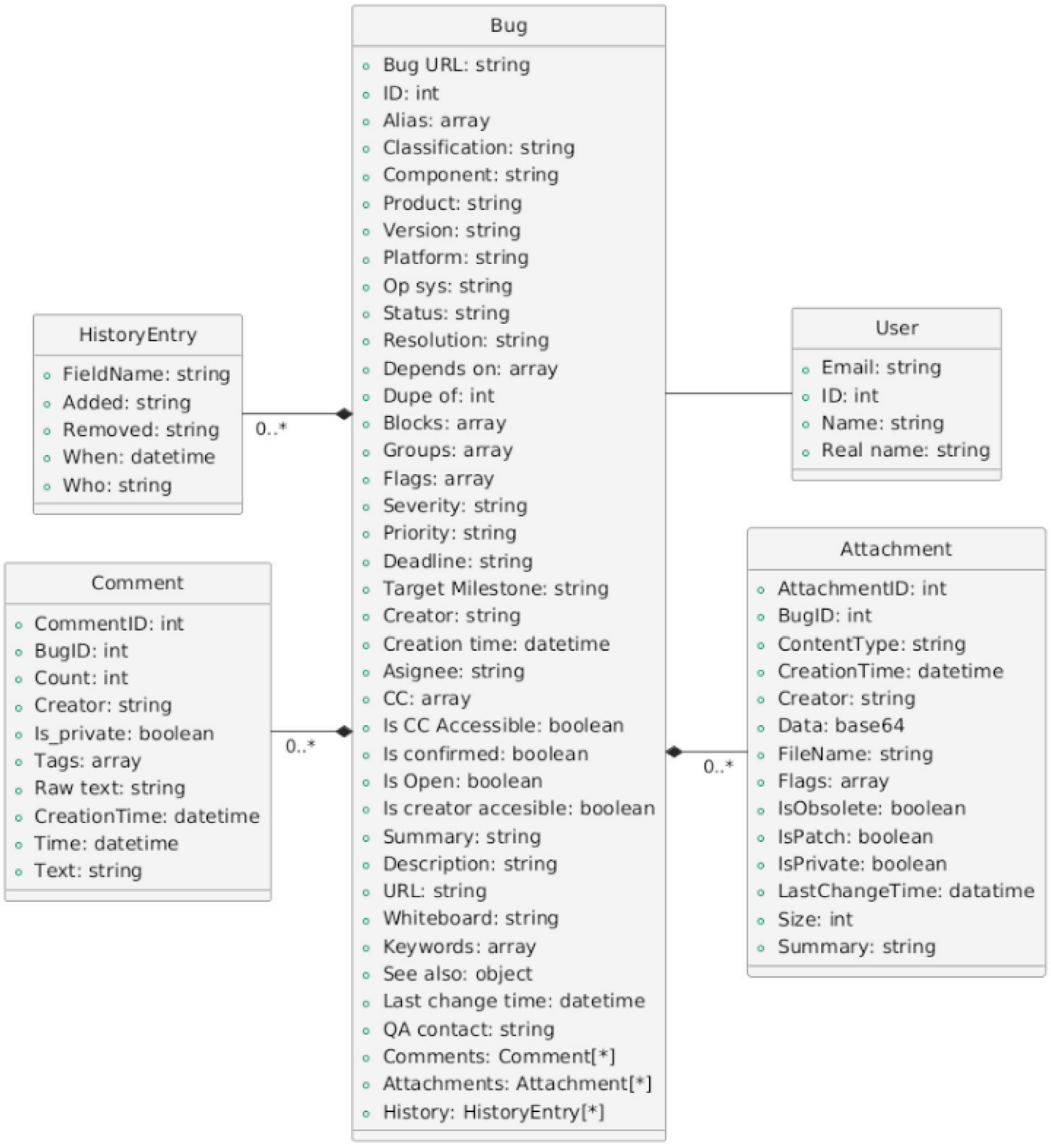
Schema of the information and type of data retrieved form bug reports.

The dataset structure (represented in Fig. 1) consists of a main directory called Eclipse, which contains all the bug reports. Each project has its own subdirectory, named after the Eclipse Product or Component where the bugs are filled. Within each subdirectory, there is a CSV file that contains all the bug-related data represented in Fig. 2.

Each **CSV file** in the dataset follows a structured format, where each line corresponds to a bug report. Every entry contains multiple attributes related to each bug report and its associated information, with all attributes and their data types detailed in Fig. 2 . The data includes but is not limited to:

**Bug data and metadata**: Bug ID, creation time (in ISO8601 format), assignee, status, resolution, severity, priority, summary, description, etc….

**Change history**: Records of modifications made to the bug, including status updates, reassignment and modifications to any other attribute.

**Comments**: This includes all developer and user comments associated with the bug report, including the author, the creation time, the content of the comment, etc.

**Attachments**: Files and logs uploaded to the Bugzilla system as supporting evidence including all the information about them such as size, summary, creator and the data itself.

The UML^2^ diagram showed in Fig. 2 offers a more detailed illustration of the various attributes and their relationships within the dataset. To complement this diagram, a sampled version of the dataset is provided in the Zenodo repository,^3^ providing an illustrative view of the data structure and fields.

Although the dataset is complete in terms of available public fields and bug reports, some attributes (such as the array of alias or keywords) are frequently empty in the original repository. In these cases, they are preserved as empty values rather than replaced by placeholders to maintain data fidelity.To summarize, the dataset provides a comprehensive collection of bug reports from key Eclipse projects, ensuring detailed historical and up-to-date information, as presented in Table 1.

**Table 1:** Number of issues by product or component.

Eclipse Product|Component	Number of bug reports
Platform	122,497
JDT	63,266
CDT	22,371
PDE	17,639
Equinox	14,559
BIRT	23,308
Mylyn	13,906
TPTP	10,579
Papyrus	13,253
TOTAL	301,378

## 4 Experimental Design, Materials and Methods

The dataset was acquired through a systematic extraction process from Bugzilla repositories. A custom Command Line Interface (CLI) based tool, Issuex, was used to automate data retrieval, ensuring a complete and structured collection of bug reports from various Eclipse projects. The tool interacts directly with Bugzilla’s REST API (validated on versions 4.3, 5.0, and 5.2) to obtain all relevant data while maintaining consistency across different Bugzilla instances. To demonstrate the adaptability of the tool and its potential for broader applications, we also provide a dataset extracted from the Mozilla Core project, showcasing its capability to retrieve and structure data from multiple Bugzilla instances.

The data collection process encompassed several steps to ensure data completeness and integrity. First, we need to identify relevant projects to the Eclipse community that are considered into its bug tracking system (Bugzilla). In this case, we decided to select them based on relevance and activity within the ecosystem. Secondly, we use the CLI tool that has two primary commands: *issuex run*, which executes the extraction process based on user-defined parameters, and *issuex run:default*, which runs the extraction using default configurations, obtaining all available bug reports regardless of their status, resolution, or creation date, without requiring user input. The tool queries Bugzilla’s API to obtain an initial list of issues, each of which is then further processed to retrieve detailed attributes, including metadata, historical changes, comments, and attachments.

Lastly, we store the results of the executions in their directory, — as shown on Fig. 1— ensuring that we follow a structure where the main folder represents the entire dataset, and the subdirectories represent each of the project (products or components). Inside those subdirectories, there is a file that contains the information about all the public bug reports, including relevant metadata such as issue status, resolution history, timestamps, assigned developers, associated comments, and attached files, ensuring a well-structured and comprehensive dataset for further analysis.

A comprehensive README file is available in the Issuex GitHub repository,^4^ providing guidance on installation, parameter customization, and adapting the tool to other Bugzilla instances.

## Limitations

Creating a comprehensive dataset from Bugzilla repositories is a complex and multifaceted task that involves various challenges and limitations. Below, we explore the challenges encountered and the inherent limitations of the dataset.

1. **Ensuring data consistency and integrity** across various Bugzilla instances has presented a significant challenge. Variations in how data is structured, recorded, and maintained across different repositories can lead to inconsistencies. To tackle this issue, we decided to obtain the information common to all Bugzilla instances, excluding any specific details related to individual repositories. Eclipse does not consider additional attributes, so the dataset presents all the available information about each bug report.
2. **Handling large volumes of data and API rate limits**. Extracting and processing large volumes of data from extensive repositories such as Eclipse and Mozilla required significant computational power and storage capacity. This challenge and variable API rate limits increased the time needed to obtain the presented dataset.
3. **Coverage of projects**. The dataset is designed to focus on key projects from Eclipse, but it does not include all products from those repositories and only includes a selection of projects that use Bugzilla.
4. **Dependency on Bugzilla's API**. The CLI-based tool depends significantly on Bugzilla's API. Although Bugzilla is not currently under active development, any changes or deprecations in the API could affect the tool's functionality.

## Ethics Statement

The authors have read and followed the ethical requirements for publication in Data in Brief and confirm that the current work does not involve human subjects, animal experiments, or any data collected from social media platforms.

## CRediT Author Statement

**Noelia Lopez-Duran:** Conceptualization, Software, Investigation, Writing - Original Draft. **David Romero-Organvidez:** Software, Validation, Writing - Review & Editing. **Fermín Cruz:** Validation, Writing - Review & Editing. **David Benavides:** Conceptualization, Writing - Review & Editing, Supervision.

## Data Availability

ZenodoMozilla Bug Report Dataset (Original data). ZenodoEclipse Bug report dataset (Original data). GithubIssuex (Original data).

## Footnotes

1 **Issuex** tool available at: https://github.com/diverso-lab/Issuex.

## Footnotes

2 https://www.uml.org.

3 https://doi.org/10.5281/zenodo.14229936.

4 https://github.com/diverso-lab/Issuex/blob/main/README.md.
